# ED_90_ of epidural esketamine with 0.075% ropivacaine for labor analgesia in nulliparous parturients: a prospective, randomized and dose-finding study

**DOI:** 10.3389/fphar.2023.1169415

**Published:** 2023-05-05

**Authors:** Siwen Lou, Qiang Du, Liwei Yu, Qingfu Wang, Jing Yu, Zhong Mei

**Affiliations:** ^1^ Department of Obstetrics, Affiliated Xiaoshan Hospital, Hangzhou Normal University, Hangzhou, China; ^2^ Department of Anesthesiology, Affiliated Xiaoshan Hospital, Hangzhou Normal University, Hangzhou, China

**Keywords:** epidural, labor analgesia, esketamine, ropivacaine, ED_90_

## Abstract

**Background:** Because it has been reported that racemic ketamine had a local anesthetic-sparing effect when used for epidural analgesia this would suggest the likelihood of a potential advantage (less pruritus) over opioid drugs. Esketamine has greater analgesic efficacy than racemic ketamine, but the optimum dosage regimen for epidural use is undetermined. The aim of this study was to determine the ED_90_ of epidural esketamine when coadministered with 0.075% ropivacaine for labor analgesia.

**Methods:** A total of 65 laboring nulliparous patients were enrolled in this study from 16 March 2022 to 15 October 2022. The patients were randomly assigned to receive 0, 0.25, 0.5, 0.75 or 1.0 mg/mL esketamine with 0.075% ropivacaine epidurally. An effective response to the epidural loading dose was defined as numerical rating scale pain score ≤3 at 30 min after the end of the epidural loading dose (10 mL of the ropivacaine 0.075% solution with the added esketamine). The ED_90_ of epidural esketamine coadministered with 0.075% ropivacaine with 95% confidence intervals for labor analgesia was determined using probit regression. Secondary outcomes and side effects were recorded.

**Results:** The estimated value of ED_90_ with 95% CIs for epidural esketamine with 0.075% ropivacaine was 0.983 (0.704–2.468) mg/mL. The characteristics of sensory and motor block, consumption of ropivacaine per hour, duration of first or second stage, Apgar scores did not differ among the five groups. The incidence of mild dizziness in Group esketamine 1.0 mg/mL was significantly higher than that in other groups (*p* < 0.05). No statistical differences were found in other side effects among groups.

**Conclusion:** The ED_90_ value of epidural esketamine coadministered with 0.075% ropivacaine for labor analgesia in nulliparous parturients was about 1.0 mg/mL. Furthermore, our results suggested that epidural esketamine would cause dose-dependent mild dizziness especially at doses up to 1.0 mg/mL. As a single epidural additive, esketamine may not be suitable for labor analgesia. Future studies may investigate the appropriate dosage of esketamine at slightly higher concentrations of local anesthetics or larger initial volume of analgesia, or explore other potential advantages of esketamine.

**Clinical Trial Registration:** (https://www.chictr.org.cn/bin/project/edit?pid=159764), identifier (ChiCTR2200057662).

## Introduction

Most labor analgesia is performed under neuraxial anaesthesia using epidural technique (Evron et al., 2004; Wilson et al., 2018). The combination use of low-dose local anesthetic and additive is routine for epidural labor analgesia (Owen et al., 2002; Lee et al., 2019). Limiting the local anesthetic dose has been advocated, with aims of reducing the occurrence of motor block, and assisted vaginal delivery (Halliday et al., 2022). Moreover, the addition of opioids reduced the dose of local anesthetics but resulted in an increase in the morbidity of pruritus (Le et al., 2001; Grangier et al., 2020). Therefore, some additives with less side effects will be used to replace opioids, to improve maternal birth experience.

The N-methyl-D-aspartate (NMDA) receptor antagonist racemic ketamine has been reported to reduce consumption of analgesics and provide better postoperative analgesia when epidurally administered (Chia et al., 1998; Choubey et al., 2017). As the S-enantiomer of ketamine, esketamine had a stronger affinity for the NMDA receptors and was twice as potent for analgesia as racemic ketamine (Pfenninger et al., 2002). Because it is likely that epidural esketamine has a local anesthetic-sparing effect this would suggest the likelihood of a potential advantage (less pruritus) over opioid drugs, however the optimum dosage regimen for epidural esketamine is undetermined.

The aim of this prospective, randomized and double-blind study was to use probit regression to determine the ED_90_ of epidural esketamine when coadministered with 0.075% ropivacaine for labor analgesia.

## Methods

### Design and study subjects

This prospective, randomized and double-blind study, Institutional Ethics Board Approval KL2022016, was registered at http://www.chictr.org.cn/edit.aspx?pid=159764&htm=4 (ChiCTR2200057662), and informed written consent was achieved from all participants. During the consent process, the patient were informed that this was an off-label use of esketamine in the neuraxial administration.

Inclusion criteria were age 18–40 years, height ≥150 cm, weight ≤100 kg, American Society of Anesthesiologists (ASA) physical status II, gestational age ≥37 weeks, and baseline NRS pain scores >3/10 (scale 0 = no pain, 10 = the worst pain). The baseline NRS pain score was defined as the mean maternal pain score for three consecutive uterine contractions prior to labor analgesia. Exclusion criteria included contraindication to epidural anaesthesia, allergy to ropivacaine or esketamine, bradycardia, pregnancy-induced hypertension or preeclampsia, refusal to participate this study.

### Study protocol

Randomization was performed by an assistant who was not further involved in the trial. The random coding sequence was done using MedCalc 18.2.1 (MedCalc Software BV, Ostend, Belgium). Subsequently, the codes were concealed in opaque sealed envelopes and randomly assigned parturients to one of five doses of esketamine with 0.075% ropivacaine: 0, 0.25, 0.5, 0.75 or 1.0 mg/mL esketamine. Another assistant, who was not involved in the follow-up study, was responsible for the preparation of the study solution. 150 mg of ropivacaine (Naropin; AstraZeneca Co., Ltd.; 75 mg/10 mL) and each esketamine dose (Aisi; Jiangsu Hengrui Co., Ltd.; 50 mg/2 mL) were mixed and diluted with normal saline to a total volume of 200 mL of study solution. Then 10 mL of the loading dose was withdrawn into the identical 10-mL syringe, and the rest 190 mL of the study solution was added to a patient-controlled epidural analgesia (PCEA) infusion pump. The attending anesthesiologist, who performed epidural anaesthesia and injected the study solution, was blinded to the dose of esketamine.

After parturients arrived in the delivery room, the pain score, non-invasive blood pressure, heart rate, pulse oximetry, and fetal heart rate were monitored. Baseline blood pressure and heart rate were recorded as the mean of 3 successive measurements after a short rest. Intravenous access was then installed into a forearm vein. Subsequently, epidural anaesthesia was performed by the attending anesthesiologist in the maternal left lateral position. After skin was infiltrated with 5 mL of 2% lidocaine, a 18G Tuohy needle was inserted at L3-4 or L2-3 vertebral interspace using the loss-of-resistance-to-air technique. A wire-reinforced epidural catheter was advanced 3–5 cm into the epidural space and then secured. Parturient were positioned supine, with left lateral uterine displacement. After the epidural catheter was aspirated and checked for blood or cerebrospinal fluid, a test dose of 3 mL of 1% lidocaine was given. And 10 mL study solution was used as a loading dose and then administered after a 3-min assessment of the test dose.

The primary outcome measure was the efficacy assessment at 30 min after the end of the epidural loading dose. An effective response to the epidural loading dose was defined as an NRS pain score ≤3. If an ineffective response was obtained, 10 mL of 1% lidocaine was administered and repeated for 15 min as requested. If the NRS pain score was still >3 after a second rescue bolus, the epidural catheter was declared misplaced and repositioned, and the patient was withdrawn from our study. After effective analgesia was achieved (NRS pain score ≤3), the electronic analgesic pump (REHN11; Jiangsu Renxian Medical Technology Co., Ltd.) was connected to the epidural catheter and initiated. The parameters of the electronic analgesia pump were set as follows: background administration rate was 3 mL/h, 10 mL bolus was administered when NRS pain score >3, and the lockout interval was 20 min. Two hours after delivery, the epidural catheter was removed and the electronic analgesic pump stopped the infusion of study solution.

The following data were recorded every 5 min until 30 min after the end of the epidural loading dose and then continuously measured every 30 min throughout labor: the pain score, non-invasive blood pressure, heart rate, oxygen saturation, and fetal heart rate. The bilateral upper sensory block to pinprick was assessed at the mid-clavicular lines. Motor block was evaluated using the modified Bromage scale: 0 = no motion; 1 = finger movement; 2 = wrist flexion against gravity force; 3 = elbow flexion against gravity force (Sane et al., 2021).

Apgar scores, onset of analgesia (defined as the duration from the end of the epidural loading dose to NRS ≤3), duration of stage of labor, total consumption dose of ropivacaine, number of patients using oxytocin augmentation and side effects (hypotension, nausea and vomiting, pruritus, bradycardia, maternal fever, respiratory depression, dizziness) were recorded. Hypotension, was defined as a 20% reduction in baseline systolic blood pressure. Bradycardia was defined as a maternal heart rate of less than 60 beats per minute. Fever was defined as maternal body temperature ≥38 °C. Respiratory depression was defined as maternal SpO_2_ < 95%. Excessive sedation was defined as Ramsay Sedation Scale value >4. Sedation was assessed using Ramsay Sedation Scale: 1 = Patient is anxious and agitated or restless, or both; 2 = Patient is co-operative, oriented, and tranquil; 3 = Patient responds to commands only; 4 = Patient exhibits brisk response to light tactile stimuli or loud auditory stimulus; 5 = Patient exhibits sluggish response to light tactile stimuli or loud auditory stimulus; 6 = Patient exhibits no response (Rasheed et al., 2019). Dizziness was assessed using grading criteria for dizziness degree: 1 = Daily life is not affected during and after dizziness attack; 2 = Daily life is forced to stop during dizziness attack, and it is recovered completely soon after dizziness attack; 3 = Patient is able to take care of most daily life after dizziness attack; 4 = Patient is not able to take care of most daily life after dizziness attack; 5 = Patient is not able to take care of all daily life after dizziness attack, and need help from others. (Mild: grade 1; Moderate: grade 2 and 3; Severe: grade 4 and 5.)

### Statistical analysis

Statistical analyses were performed with SPSS 25.0 for Windows (IBM Corp, Armonk, NY). All data were assessed for normal distribution using the Shapiro-Wilk test and presented as mean ± SD or median with quartiles. Normally distributed data were analyzed using one-way ANOVA. Non-normally distributed data were analyzed using the Mann-Whitney U test. Frequency data were analyzed using χ2 test or Fisher’s exact test. A *p*-value <0.05 was considered statistically significant.

Based on data from our pre-experiment (Institutional Ethics Board Approval number: KL2022016Pre-trial), the percentages of patients with effective labor analgesia were 0.3, 0.4, 0.5, 0.7, and 0.9 in patients (10 per subgroup) who received epidural esketamine at doses of 0, 0.25, 0.5, 0.75, and 1.0 mg/mL with 0.075% ropivacaine respectively. A sample of 11 patients per group was required, which would provide a power of 0.90 with a significance level of 0.05 (PASS 11, NSCC, LCC, Kaysville, UT: Cochran-Armitage test for trend in proportions). To account for dropouts, we increased the sample size to 13 for each dose group.

The primary endpoint was the efficacy assessment at 30 min after the end of the epidural loading dose. An effective response to the epidural loading dose was defined as an NRS pain score ≤3. The value for ED_90_ of epidural esketamine when coadministered with 0.075% ropivacaine for labor analgesia was determined using probit regression. The Kaplan-Meier survival curve or Cox proportional hazard regression model was used to analyze the onset of analgesia of each group. In this study, equivalent dose conversion was performed to convert the lidocaine dose, including the trial dose and the subsequent additional dose, to the ropivacaine dose, the potency of ropivacaine to lidocaine is approximately 3:1 (Polley LS et al., 1999; Miller, R.D., 2014). Then, the difference in local anesthetic-sparing effect among groups was assessed by calculating consumption of ropivacaine per hour (duration from the end of the epidural loading dose to 2 h after delivery).

## Results

**FIGURE 1:**
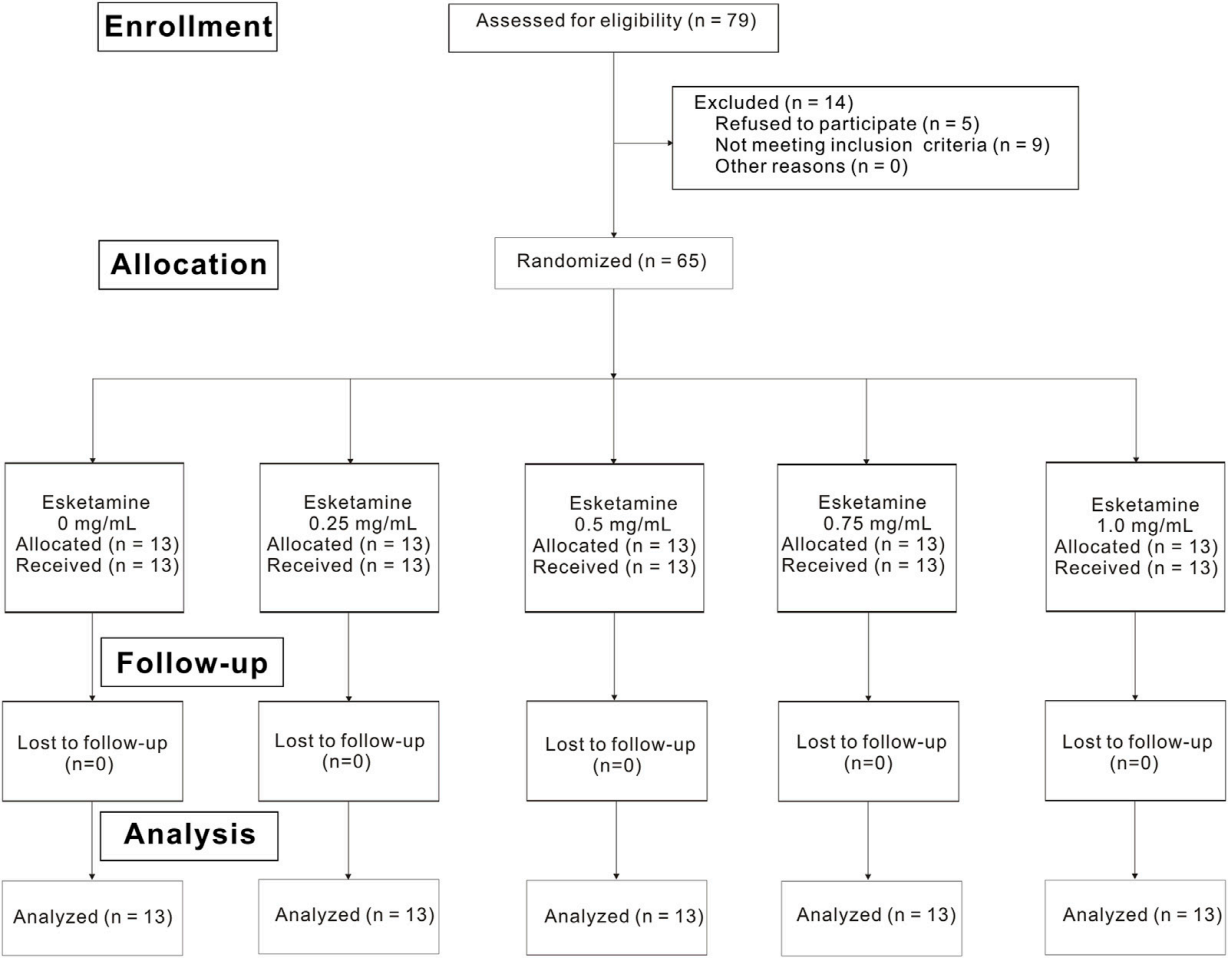
Flowchart of participants.

Of the 79 nulliparous patients available for the study, 14 patients were excluded. Consequently, a total of 65 laboring nulliparous parturients with singleton pregnancy were included in the final statistical analysis (Figure 1). Demographic data are summarized in Table 1. There were no statistically differences in age, height, weight, gestational age, cervical dilation and NRS before epidural among five groups. Secondary outcomes are shown in Table 2. No differences were found in the characteristics of sensory and motor block, duration of first stage, duration of second stage, consumption of ropivacaine per hour, proportion of spontaneous labor, induced labor, cesarean delivery and using oxytocin augmentation, 1 min Apgar score and 5 min Apgar score among groups. Number of lidocaine use differed among groups.

**TABLE 1:** Demographic data.

	Esketamine 0 mg/mL (n = 13)	Esketamine 0.25 mg/mL (n = 13)	Esketamine 0.5 mg/mL (n = 13)	Esketamine 0.75 mg/mL (n = 13)	Esketamine 1 mg/mL (n = 13)	*p*-Value
Age (years)	28.1 ± 2.5	27.2 ± 2.9	27.3 ± 1.8	27.5 ± 2.1	27.6 ± 2.5	0.908
Height (cm)	162.9 ± 3.9	158.8 ± 4.7	162.0 ± 3.8	163.0 ± 5.4	161.0 ± 4.4	0.124
Weight (kg)	71.3 ± 5.7	65.3 ± 9.1	69.0 ± 7.9	70.7 ± 10.3	65.6 ± 6.5	0.200
Gestational age (weeks)	39.2 ± 1.2	39.2 ± 0.8	39.2 ± 0.8	38.9 ± 1.0	39.1 ± 1.0	0.809
Cervical dilation (cm)	2.8 ± 0.6	2.9 ± 0.6	2.9 ± 0.5	2.5 ± 0.5	2.8 ± 0.4	0.311
NRS before epidural	7 (6.7)	7 (7.8)	7 (6.8)	7 (6.7)	6 (6.8)	0.240

Data are mean ± SD (standard deviation), or median (interquartile range) or number (%).

**TABLE 2:** Secondary outcomes.

	Esketamine 0 mg/ml (*n* = 13)	Esketamine 0.25 mg/ml (*n* = 13)	Esketamine 0.5 mg/ml (*n* = 13)	Esketamine 0.75 mg/ml (*n* = 13)	Esketamine 1 mg/ml (*n* = 13)	*P* value
Effective rate	5 (38%)	4 (31%)	7 (54%)	11 (85%)	12 (92%)	0.003
Receiving lidocaine administration	8 (62%)	9 (69%)	6 (46%)	2 (15%)	1 (8%)	0.003
Number of lidocaine use	1 (0–1)	1 (0–1)	0 (0–1)	0 (0–0)	0 (0–0)	0.005
Sensory block level (pinprick)	T9 (9–10)	T9 (8–10)	T10 (9–10)	T10 (8–10)	T9 (8–10)	0.253
Bromage score >0	0 (0%)	0 (0%)	0 (0%)	0 (0%)	0 (0%)	>0.999
Duration of first stage (minutes)	613.2 ± 152.7	586.2 ± 186.2	547.7 ± 126.5	498.8 ± 202.2	535.4 ± 184.9	0.490
Duration of second stage (minutes)	61.9 ± 54.9	41.5 ± 35.5	43.2 ± 25.4	63.2 ± 37.0	78.2 ± 41.6	0.121
Consumption of ropivacaine per hour (mL)	13.9 ± 5.4	13.3 ± 4.7	13.2 ± 3.6	12.0 ± 4.1	13.2 ± 5.5	0.881
Using oxytocin augmentation	1 (8%)	3 (23%)	1 (8%)	2 (15%)	3 (23%)	0.676
Spontaneous labor	12 (92%)	9 (69%)	11 (85%)	8 (62%)	8 (62%)	0.215
Induced labor	1 (8%)	2 (15%)	2 (15%)	3 (23%)	5 (38%)	0.366
Cesarean delivery rate	0 (0%)	2 (15%)	0 (0%)	2 (15%)	0 (0%)	0.101
Apgar score, 1 min	10 (10–10)	10 (10–10)	10 (10–10)	10 (10–10)	10 (10–10)	>0.999
Apgar score, 5 min	10 (10–10)	10 (10–10)	10 (10–10)	10 (10–10)	10 (10–10)	>0.999

Data are mean ± SD (standard deviation), or median (interquartile range) or number (%).

**FIGURE 2:**
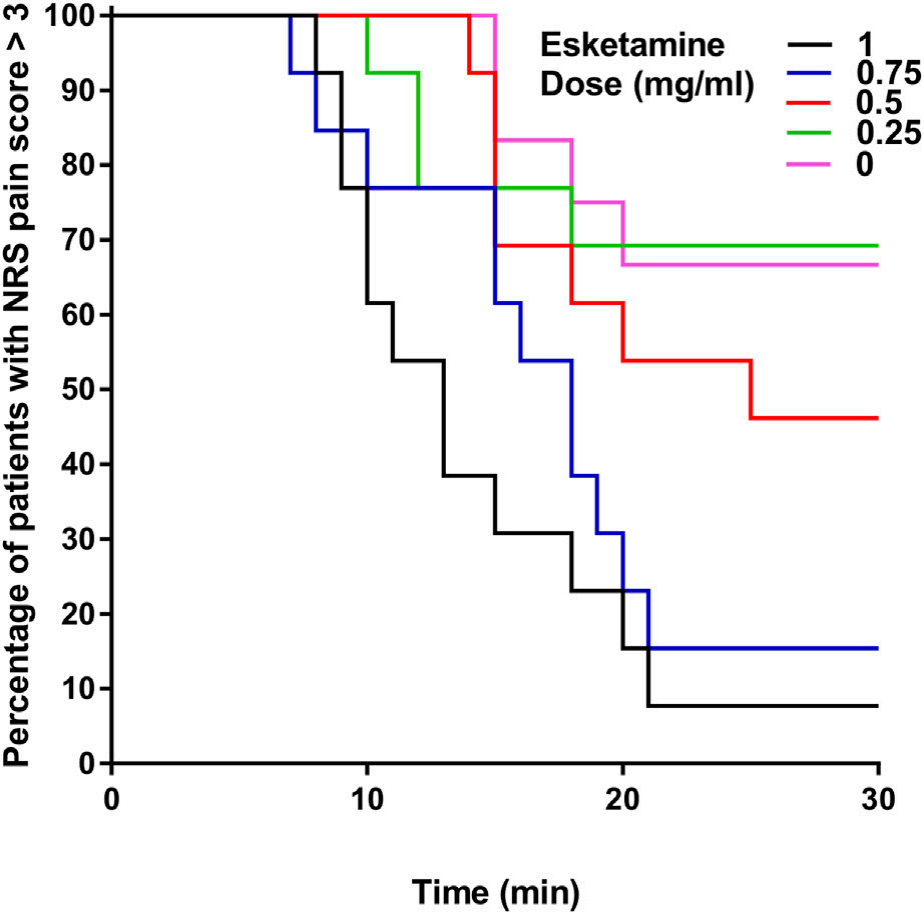
Kaplan–Meier survival curves showing the percentage of patients with NRS pain score >3. An ineffective response to epidural loading dose was defined as an NRS pain score >3 at 30 min after the end of epidural loading dose.

**FIGURE 3:**
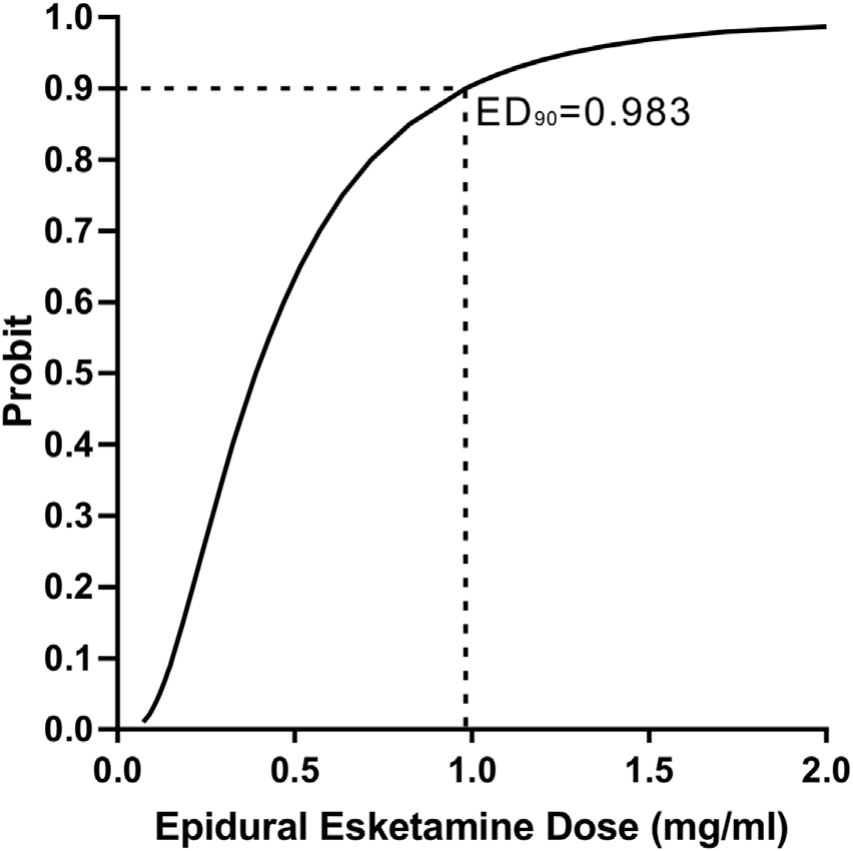
Probit regression curve for effective epidural labor analgesia. An effective response to the epidural loading dose was defined as an NRS pain score ≤3 at 30 min after the end of the epidural loading dose.

There were 8, 9, 6, 2 and 1 patients with ineffective analgesia in the 0-, 0.25-, 0.5-, 0.75- and 1.0- mg/mL groups according to the definition, and there was no statistically difference in survival curve distribution of onset of analgesia among groups (*p* = 0.388) (Figure 2). Labor analgesia was effective in 38, 31, 54, 85% and 92% of the 0-, 0.25-, 0.5-, 0.75- and 1.0- mg/mL groups, respectively. Using probit regression the ED_90_ of epidural esketamine coadministered with ropivacaine for labor analgesia were determined. The estimated value of ED_90_ with 95% CIs for epidural esketamine with 0.075% ropivacaine was 0.983 (0.704–2.468) mg/mL (Figure 3).

No side effects such as hypotension, nausea and vomiting, pruritus, bradycardia, maternal fever, respiratory depression and excessive sedation occurred in each subgroup, and there were no significant differences in these side effects among groups. The incidence of mild dizziness in Group esketamine 1.0 mg/mL was significantly higher than that in other groups (*p* < 0.001) (Table 3).

**TABLE 3:** The occurrence of dizziness.

	Esketamine 0 mg/mL (n = 13)	Esketamine 0.25 mg/mL (n = 13)	Esketamine 0.5 mg/mL (n = 13)	Esketamine 0.75 mg/mL (n = 13)	Esketamine 1.0 mg/mL (n = 13)	*p*-Value
Dizziness						
Mild	0*	0*	0*	4*	12	<0.001*
Moderate	0	0	0	0	0	>0.999
Severe	0	0	0	0	0	>0.999

Data are numbers.

*p < 0.05 for comparison with 1.0 mg/mL value using Fisher’s exact test.

## Discussion

Our study suggested the value for ED_90_ of epidural esketamine with 0.075% ropivacaine for labor analgesia in nulliparous parturients was approximately 1.0 mg/mL. Additionally, our results suggested that epidural esketamine would cause dose-dependent mild dizziness, especially when the dose reached 1.0 mg/mL.

In present study, for the cases with ineffective analgesia in each group, the addition of lidocaine would certainly have an impact on the consumption of ropivacaine. Therefore, the lidocaine dose, including the trial dose and the subsequent additional dose, was converted to the ropivacaine dose. Ultimately, we did not find a statistical difference in the consumption of ropivacaine per hour among groups.

We did not find epidural esketamine to have an anesthetic-sparing effect, which is similar to previous work reported that epidural ketamine did not reduce analgesic consumption (Gottschalk et al., 2008; Joseph et al., 2012). However, Chia YY et al. demonstrated that adding ketamine to morphine and bupivacaine reduced the hourly consumption of local anesthetic (Chia et al., 1998). The difference between these studies is uncertain but may be related to the synergistic analgesic effect provided by epidural morphine. Additionally, the lack of difference in ropivacaine use per hour in our study might have something to do with this PCEA protocol. The background rate of only 3 mL/h (2 mg ropivacaine) was so low that almost all patients needed 1–2 patient-controlled boluses of this dilute solution every hour, but it seemed that a recipe more like 6–8 mL/h, with 5–6 mL PCEA boluses, would be more likely to demonstrate a difference (Stratmann et al., 2005; Gupta et al., 2016).

Our study results suggested the effective rate of epidural labor analgesia increased with the increasement of esketamine dose, this may be due to the powerful analgesic effect of esketamine itself and the blockade of NMDA receptors in the spinal cord. But epidural esketamine resulted in dose-dependent mild dizziness, especially at doses up to 1.0 mg/mL. Therefore, esketamine may be unsuitable as a single additive to local anesthetics for epidural labor analgesia. Nevertheless, low-dose esketamine was still recommended as one of the combined additives for epidural analgesia because of its potential to prevent opioid tolerance and block central hypersensitive states (Dickenson et al., 1997; Kido et al., 2019).

A predictable finding was that the incidence of maternal motor block was 0% when 0.075% ropivacaine was used for epidural labor analgesia. 0.075% ropivacaine concentration might be very low, as many studies suggest that ropivacaine is 20%–40% less potent than bupivacaine, and 0.075% ropivacaine is as effective as 0.05%–0.0625% bupivacaine in labor analgesia (Ngan Kee et al., 2010; Wang et al., 2010). Ultra-low (≤0.08%) concentrations of local anesthetics were associated with an incremental likelihood of spontaneous labor, reduced motor block, and shorter the duration of second stage (Halliday et al., 2022).

Previous studies have shown that low concentration and high volume of local anesthetic with additives have better analgesic effects than high concentration local anesthetic (Owen et al., 2002; Halliday et al., 2022). With the advantages of good analgesic effect potency, and extended effective analgesia time, opioids have been widely used in labor analgesia (Dostbil et al., 2014; Wen et al., 2021). However, the incidence of opioids-associated side effects (e.g., pruritus) will increase (Le et al., 2001; Grangier et al., 2020). Alpha 2 agonists clonidine or dexmedetomidine can provide excellent labor analgesia, but they may prolong the second stage of labor (Kabi et al., 2021; Ni et al., 2022). In this study the addition of single esketamine to ropivacaine caused maternal dizziness, low-dose esketamine in combination with other additives such as morphine may produce a more ideal analgesic effect in labor.

Our study has several limitations. First of all, this study used 10 mL of 0.075% ropivacaine as a loading dose, and the ED_90_ of esketamine would certainly have been different if 10 mL of 0.1% ropivacaine or 15 mL of 0.075% ropivacaine had been used. Therefore, our results may not apply to different concentrations or volumes of ropivacaine used as loading doses for epidural labor analgesia. A follow-up study was conducted to explore this question. Secondly, in contrast to patient controlled epidural analgesia (PCEA) technique we used, programmed intermittent epidural bolus (PIEB) had superior analgesic quality (Roofthooft et al., 2020). It is not clear whether our results can be applied to the PIEB technique. Thirdly, NRS≤3 is not a particularly “high bar” for labor analgesia - most studies seem to use ≤1 to show efficacy. This would likely have required more ropivacaine, since the loading dose was only 7.5 mg, well below the expected ED_50_ of 18 mg in two classic up-down sequential allocation studies (Benhamou et al., 2003; Polley et al., 2003). Fourthly, it was probably not a great choice to include this test dose (3 mL 1% lidocaine) in the protocol. It is a small, but not completely insignificant amount of local anesthetic, so would provide some degree of analgesia, would tend to decrease any differences between the study groups. It is also not a particularly effective test dose anyway, containing no epinephrine to elicit a tachycardia, although probably would suggest a intrathecal catheter (Pratt et al., 2013). Fifthly, our study was underpowered for almost all of the secondary outcomes because of the small number in each subgroup (n = 13). Sixthly, our study was in the absence of control group in which low dose of opioid would be added to the solution of local anesthetic for labor epidural analgesia, as well as group in which low dose of opioid would be combined with esketamine (both added to local anesthetic solution). We will conduct a comparative study on the efficacy of these drugs for epidural labor analgesia. Lastly, as we only enrolled nulliparous parturients, we do not know whether our results apply to multiparae.

In conclusion, the ED_90_ value of epidural esketamine coadministered with 0.075% ropivacaine for labor analgesia in nulliparous parturients was about 1.0 mg/mL. Furthermore, our results suggested that epidural esketamine would cause dose-dependent mild dizziness especially at doses up to 1.0 mg/mL. As a single epidural additive, esketamine may not be suitable for labor analgesia. Future studies may investigate the appropriate dosage of esketamine at slightly higher concentrations of local anesthetics or larger initial volume of analgesia, or explore other potential advantages of esketamine.

## Data Availability

The raw data supporting the conclusions of this article will be made available by the authors, without undue reservation.

## Abbreviations

NRS pain scores, numerical rating scale pain scores; ED_90_, the 90% effective dose; CIs, confidence intervals; N-methyl-D-aspartate receptor antagonist, NMDA receptor antagonist; ASA, American Society of Anesthesiologists; PCEA, patient controlled epidural analgesia; ANOVA, analysis of variance; PIEB, programmed intermittent epidural bolus.
